# Dose-dependent effects of the once-daily GLP-1 receptor agonist lixisenatide in patients with Type 2 diabetes inadequately controlled with metformin: a randomized, double-blind, placebo-controlled trial

**DOI:** 10.1111/j.1464-5491.2010.03020.x

**Published:** 2010-09

**Authors:** R E Ratner, J Rosenstock, G Boka, on behalf of the DRI6012 Study Investigators

**Affiliations:** Medstar Research Institute and Georgetown University Medical SchoolWashington, DC, USA; * Dallas Diabetes and Endocrine Center at Medical CityDallas, TX, USA; † sanofi-aventis R&DChilly-Mazarin cedex, France

**Keywords:** glucagon-like peptide-1 receptor agonist, glycaemic control, lixisenatide, Type 2 diabetes

## Abstract

**Aims:**

To evaluate the dose–response relationship of lixisenatide (AVE0010), a glucagon-like peptide-1 (GLP-1) receptor agonist, in metformin-treated patients with Type 2 diabetes.

**Methods:**

Randomized, double-blind, placebo-controlled, parallel-group, 13 week study of 542 patients with Type 2 diabetes inadequately controlled [glycated haemoglobin (HbA_1c_) ≥ 7.0 and < 9.0% (≥ 53 and < 75 mmol/mol)] on metformin (≥ 1000 mg/day) treated with subcutaneous lixisenatide doses of 5, 10, 20 or 30 μg once daily or twice daily or placebo. The primary end-point was change in HbA_1c_ from baseline to 13 weeks in the intent-to-treat population.

**Results:**

Lixisenatide significantly improved mean HbA_1c_ from a baseline of 7.55% (59.0 mmol/mol); respective mean reductions for 5, 10, 20 and 30 μg doses were 0.47, 0.50, 0.69 and 0.76% (5.1, 5.5, 7.5 and 8.3 mmol/mol), on once-daily and 0.65, 0.78, 0.75 and 0.87% (7.1, 8.5, 8.2 and 9.5 mmol/mol) on twice-daily administrations vs. 0.18% (2.0 mmol/mol) with placebo (all *P*< 0.01 vs. placebo). Target HbA_1c_ < 7.0% (53 mmol/mol) at study end was achieved in 68% of patients receiving 20 and 30 μg once-daily lixisenatide vs. 32% receiving placebo (*P*< 0.0001). Dose-dependent improvements were observed for fasting, postprandial and average self-monitored seven-point blood glucose levels. Weight changes ranged from −2.0 to −3.9 kg with lixisenatide vs. −1.9 kg with placebo. The most frequent adverse event was mild-to-moderate nausea.

**Conclusions:**

Lixisenatide significantly improved glycaemic control in mildly hyperglycaemic patients with Type 2 diabetes on metformin. Dose–response relationships were seen for once- and twice-daily regimens, with similar efficacy levels, with a 20 μg once-daily dose of lixisenatide demonstrating the best efficacy-to-tolerability ratio. This new, once-daily GLP-1 receptor agonist shows promise in the management of Type 2 diabetes to be defined further by ongoing long-term studies.

## Introduction

Glycaemic control in Type 2 diabetes mellitus is generally targeted toward a glycated haemoglobin (HbA_1c_) level as close to normal [i.e. < 6.5 or < 7.0% (< 48 or < 53 mmol/mol)] as safely as possible [1,2]. Although a variety of pharmacological approaches are now available, current management often fails to achieve glycaemic targets [3]. Analogues of the hormone glucagon-like peptide-1 (GLP-1) have shown promise as therapeutic options in Type 2 diabetes. Endogenous GLP-1 enhances insulin secretion and inhibits postprandial glucagon secretion in a glucose-dependent fashion, slows gastric emptying, reduces food intake and promotes weight loss, with all these effects matched by GLP-1 receptor agonists [4,5]. The suppression of glucagon by GLP-1 does not occur at hypoglycaemic glucose levels, and as such exogenous GLP-1 does not impair the physiological mechanisms that counteract hypoglycaemia [6].

Pharmacological replacement with GLP-1 receptor agonists in patients with Type 2 diabetes represents an attractive strategy to improve metabolic control, particularly as we aim for near-normal glycaemia, with less risk of hypoglycaemia and no weight gain or, ideally, weight loss. In addition to improving glycaemic control, GLP-1 receptor agonists have the potential to preserve pancreatic islet B cells by enhancing proliferation and inhibiting apoptosis, based on preclinical studies in animal models and cultured pancreatic B cells [7–12], but still with no evidence in human studies.

However, exogenous native GLP-1 is not suitable as a therapeutic agent because it is rapidly degraded by dipeptidyl peptidase-4 (DPP-4) and has a half-life of less than 2 min [4]. Thus, DPP-4-resistant GLP-1 receptor agonists with extended half-lives have been developed. Lixisenatide (AVE0010) is a new, potent, selective and synthetic 44 amino acid exendin-4-like GLP-1 receptor agonist modified C-terminally with six Lys residues and one Pro deleted. In Chinese hamster ovary (CHO) cells transfected with the human GLP-1 receptor, lixisenatide had a binding affinity approximately 4-fold greater than that of native human GLP-1 (IC_50_ for lixisenatide = 1.43 nmol/l vs. IC_50_ for GLP-1 = 5.48 nmol/l) [9,13]. Lixisenatide is being developed with the aim of improving the management of Type 2 diabetes. The primary objective of this study was to evaluate thoroughly the dose–response effect of lixisenatide using once- or twice-daily regimens (5–30 μg once or twice daily) on HbA_1c_ changes over 13 weeks in metformin-treated patients with Type 2 diabetes.

## Patients and methods

### Study participants

The study population comprised male and female patients aged 30–75 years with Type 2 diabetes mellitus of at least 1 year’s duration inadequately controlled [HbA_1c_≥ 7.0 and < 9.0% (≥ 53 and < 75 mmol/mol)] on stable metformin monotherapy (≥ 1000 mg/day) for at least 3 months prior to screening.

The main exclusion criteria were as follows: history of gastrointestinal disease with prolonged nausea and vomiting during the previous 6 months; history of chronic pancreatitis or stomach/gastric surgery; severe cardiovascular events during the previous 6 months; or hepatic or renal disease at screening [serum creatinine ≥ 114.4 μmol/l (1.5 mg/dl) for males and ≥ 106.8 μmol/l (1.4 mg/dl) for females].

The study was approved by the institutional review boards or ethics committees and was conducted in accordance with the Declaration of Helsinki and Good Clinical Practice guidelines. All patients gave written informed consent to participate in the study.

### Study design

This 13 week, multinational, randomized, parallel-group, placebo-controlled study was conducted at 133 centres between March 2006 and August 2007. The study drug, added-on to stable metformin, was double-blind regarding active treatment or placebo and open-label regarding the treatment volume.

Following a 2 week screening phase, eligible patients entered into a 2 week, single-blind, placebo run-in period. Eligible patients were then randomized at visit 4 (week 0) to one of 12 treatment arms (2:2:2:2:2:2:2:2:1:1:1:1): to subcutaneous injec-tions of lixisenatide doses of 5, 10, 20 or 30 μg administered once daily within 1 h before breakfast (with volume-matched placebo before dinner); to lixisenatide doses of 5, 10, 20 or 30 μg administered twice daily (10, 20, 40 or 60 μg total daily dose, respectively) within 1 h before both breakfast and dinner; or to one of four volume-matched placebo treatments administered twice daily.

Randomization of subjects, allocation of medication and management of drug supplies were performed using an interactive voice response system.

Dose escalation was performed during the first 2–4 weeks for patients randomized to 20 and 30 μg dose levels of the study medication; the dose was initiated at 10 μg for 1 week and increased by 5 μg/week up to the target dose. The entry dosage of metformin remained unchanged throughout the study. All patients received diet and lifestyle counselling according to the American Diabetes Association guidelines [1].

### Study assessments

The primary efficacy end-point was change in HbA_1c_ from baseline to study end for the intent-to-treat population. Glycated haemoglobin was measured at a National Glycohemoglobin Standardization Program (NGSP) Level 1 certified central laboratory, measured with the high-performance liquid chro-matography method. Corresponding International Federation of Clinical Chemistry and Laboratory Medicine (IFCC) standardized values were calculated using the relationship: IFCC value (in mmol/mol) = (NGSP value – 2.152)/0.09148 [14,15]. All HbA_1c_ data are given as NGSP standardized values and IFCC values. The secondary efficacy measures included the percentage of patients achieving an HbA_1c_ < 7.0 or < 6.5% (< 53 or < 48 mmol/mol), changes in body weight, fasting plasma glucose, and 2 h post-prandial plasma glucose after a standardized breakfast. Self-monitored seven-point blood glucose measurements were performed at baseline and week 13. Anti-lixisenatide antibody levels were measured.

Safety and tolerability were assessed by physical examination, adverse event reporting, blood pressure, heart rate, 12-lead electrocardiogram and standard laboratory measurements. Symptomatic hypoglycaemia was defined as symptoms consis-tent with hypoglycaemia, with an accompanying blood glucose < 3.3 mmol/l or prompt recovery with carbohydrate.

### Statistical analyses

Sample sizes of 50 patients in each active treatment group and 100 patients in the placebo group were calculated to provide a statistical power of 81% to detect a 0.6% (6.6 mmol/mol) difference in HbA_1c_ between an active treatment and placebo assuming a standard deviation of 1.2% (13.1 mmol/mol). Statistical significance was assumed at the 5% level.

Analyses of the primary efficacy variable (changes in HbA_1c_ from baseline to end-point) were performed using analysis of covariance (ANCOVA) model, with treatment and country as fixed factors and baseline HbA_1c_ as a covariate. Multiple testing procedure was used for the primary efficacy variable in order to control Type 1 error for the study and for multiple doses within each dose regimen (once and twice daily). The step-down trend test was used from the above ANCOVA model to assess dose–response relationship within each regimen. The continuous secondary efficacy variables (change in body weight, fasting plasma glucose, seven-point self monitored blood glucose and post-prandial plasma glucose) were analysed using the same methods used for the primary efficacy variable. Data from placebo-treated subjects were pooled for statistical analysis. Both means and least square adjusted means were calculated. The percentages of patients achieving an HbA_1c_ < 7.0 and < 6.5% (< 53 and < 48 mmol/mol) were analysed using a Cochran–Mantel–Haenszel test stratified by country. Safety and tolerability data were analysed using descriptive statistics for all patients who received at least one dose of study medication.

Unless otherwise indicated, all efficacy data were analysed in the intent-to-treat population (all randomized subjects taking at least one dose of the study medication and having a baseline and one on-treatment value for efficacy variables); they are presented as means ± sem, unless specified otherwise.

## Results

### Demographic and baseline characteristics

A total of 542 patients were randomized from 1466 patients screened. The reasons for screening failure (*n* = 924) were as follows: ineligible inclusion criteria (*n* = 850), patient’s wish (*n* = 34) and other (*n* = 40). Approximately 90% of patients completed 13 weeks of active treatment, and the percentages ranged from 83% in the 30 μg lixisenatide once daily group to 96% in the 5 μg lixisenatide once daily and twice daily groups, compared with 95% in the placebo group (Table 1). Nearly all patients were at their randomized dose level by study end, ranging from 85 and 89% in the 30 μg once daily and twice daily groups to 100% in the 5 μg once daily and twice daily and 10 μg twice daily groups.

**Table 1:** Patient disposition, demographics and baseline characteristics (safety population)

		Lixisenatide							
		
	Placebo	5 μg QD	10 μg QD	20 μg QD	30 μg QD	5 μg BID	10 μg BID	20 μg BID	30 μg BID
Patient disposition, *n* (%)									
Randomized	109	55	52	55	54	53	56	54	54
Completed	103 (94.5)	53 (96.4)	47 (90.4)	46 (83.6)	45 (83.3)	51 (96.2)	51 (91.1)	46 (85.2)	47 (87.0)
Discontinued	6 (5.5)	2 (3.6)	5 (9.6)	9 (16.4)	9 (16.7)	2 (3.8)	5 (8.9)	8 (14.8)	7 (13.0)
Reasons for discontinuation, *n* (%)									
Adverse event	2 (1.8)	1 (1.8)	2 (3.8)	3 (5.5)	6 (11.1)	0	2 (3.6)	8 (14.8)	5 (9.3)
Lack of efficacy	0	1 (1.8)	0	0	1 (1.9)	0	0	0	0
Other	4 (3.7)	0	3 (5.8)	6 (10.9)	2 (3.7)	2 (3.8)	3 (5.4)	0	2 (3.7)
Demographics and baseline characteristics									
Mean age (years ± sd)	56.3 ± 9.2	56.8 ± 7.8	55.4 ± 9.2	55.4 ± 9.9	56.5 ± 8.7	57.1 ± 8.2	56.0 ± 7.9	56.7 ± 8.3	55.3 ± 9.1
Male, *n* (%)	61 (56.0)	26 (47.3)	31 (59.6)	28 (50.9)	27 (50.0)	25 (47.2)	29 (51.8)	20 (37.0)	23 (42.6)
Race, *n* (%)									
Caucasian	84 (77.1)	38 (69.1)	36 (69.2)	45 (81.8)	43 (79.6)	46 (86.8)	45 (80.4)	42 (77.8)	35 (64.8)
Black	12 (11.0)	5 (9.1)	6 (11.5)	1 (1.8)	6 (11.1)	2 (3.8)	2 (3.6)	2 (3.7)	9 (16.7)
Other	13 (11.9)	12 (21.8)	10 (19.3)	9 (16.4)	5 (9.3)	5 (9.4)	9 (16.0)	10 (18.5)	10 (18.5)
Mean duration of diabetes diagnosis (years ± sd)	7.1 ± 5.4	7.2 ± 4.9	6.2 ± 4.1	6.4 ± 6.8	6.0 ± 4.8	6.2 ± 6.0	6.4 ± 5.0	6.6 ± 5.1	7.0 ± 5.4
Mean HbA_1c_ (% ± sd; NGSP)	7.53 ± 0.6	7.58 ± 0.7	7.52 ± 0.6	7.58 ± 0.7	7.52 ± 0.7	7.60 ± 0.6	7.54 ± 0.6	7.61 ± 0.7	7.46 ± 0.5
Mean HbA_1c_ (mmol/mol ± sd; IFCC)*	58.8 ± 5	59.3 ± 6	58.7 ± 5	59.3 ± 6	58.7 ± 6	59.6 ± 5	58.9 ± 5	59.7 ± 6	58.0 ± 4
Mean weight (kg ± sd)	87.7 ± 14	84.6 ± 16	90.5 ± 17	89.4 ± 17	87.6 ± 15	86.5 ± 14	89.8 ± 17	88.5 ± 17	87.5 ± 14
Mean BMI (kg/m^2^ ± sd)	31.7 ± 4.2	30.7 ± 4.6	31.9 ± 4.0	32.0 ± 4.3	31.6 ± 3.6	31.6 ± 4.2	32.8 ± 4.4	32.7 ± 4.4	32.3 ± 4.5
Mean fasting plasma glucose (mmol/l ± sd)	8.8 ± 2.1	8.4 ± 2.1	8.7 ± 2.0	8.4 ± 1.8	8.9 ± 2.1	8.9 ± 1.8	9.2 ± 2.4	9.0 ± 2.0	8.8 ± 2.3
Mean 2 h post prandial plasma glucose (mmol/l ± sd)	11.8 ± 3.2	12.8 ± 2.4	12.8 ± 3.3	11.5 ± 2.5	13.1 ± 3.3	12.5 ± 2.6	12.0 ± 3.3	12.3 ± 2.7	12.2 ± 3.0

Abbreviations: IFCC, International Federation of Clinical Chemistry and Laboratory Medicine; NGSP, National Glycohemoglobin Standardization Program; Postprandial plasma glucose measured in a subgroup of patients undergoing standardized breakfast in selected sites. The standardized 500 kcal breakfast, which consisted of orange juice (180 ml), toasted bread (60 g), jam or preserves (20 g), butter or margarine (10 g), whole milk (120 ml) and coffee or tea with non-nutritive sweetener (if desired), was consumed within a 15 min period. The safety population consisted of all randomized patients who took at least one dose of the study medication during the double-blind treatment phase.

* Calculated IFCC standardized HbA_1c_ value (in mmol/mol) = (NGSP value – 2.152)/0.09148 [14,15].

Demographic and baseline characteristics were well matched, and there were no clinically relevant differences between groups (Table 1).

### Efficacy

**FIGURE 1:**
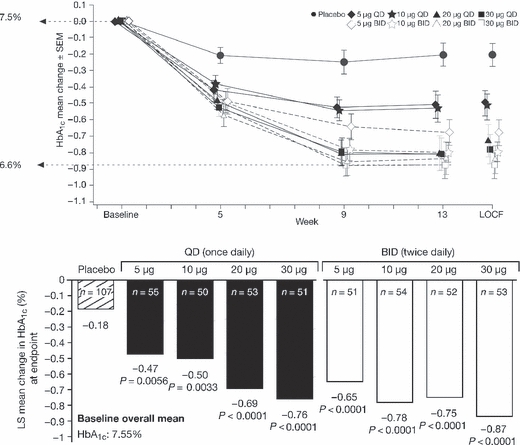
Changes in glycated haemoglobin (HbA_1c_) levels following 13 weeks' treatment with lixisenatide once daily or twice daily, according to dosage and regimen. Top panel shows change in mean (±sem) HbA_1c_ over time. Bottom panel shows least square (LS) mean change in HbA_1c_ from baseline to 13 weeks.

There were significantly greater improvements in the primary efficacy end-point of HbA_1c_ change from a mean overall baseline of 7.55% (59.0 mmol/mol) in all the lixisenatide groups (*P* < 0.01 vs. placebo), with reductions ranging from 0.47 to 0.87% (from 5.1 to 9.5 mmol/mol) among the different dosing regimens (mean reductions for 5, 10, 20 and 30 μg doses of 0.47, 0.50, 0.69 and 0.76% [5.1, 5.5, 7.5 and 8.3 mmol/mol] on once-daily administration, respectively, and 0.65, 0.78, 0.75 and 0.87% (7.1, 8.5, 8.2 and 9.5 mmol/mol) on twice-daily administration, respectively), compared with a decrease of 0.18% (2.0 mmol/mol) for placebo (Fig. 1). A dose–response relationship with HbA_1c_ level was seen for both the once daily and twice daily regimens of lixisenatide, with improvements in HbA_1c_ observed as early as week 5 (Fig. 1).

**FIGURE 2:**
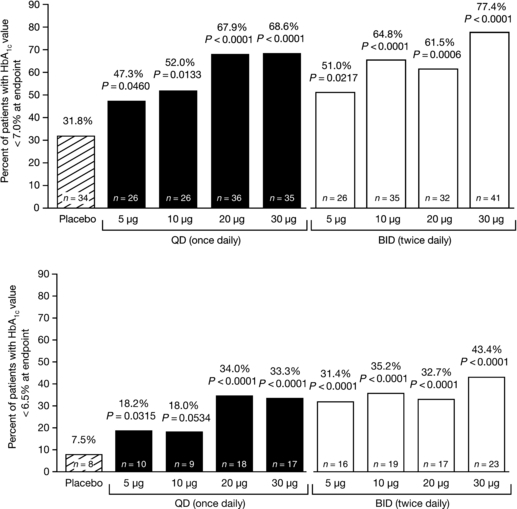
Percentage of patients with glycated haemoglobin (HbA_1c_) level of < 7.0% (53 mmol/mol; top panel) and < 6.5% (48 mmol/mol; bottom panel) following 13 weeks’ treatment with lixisenatide once daily or twice daily, according to dosage and regimen.

Significantly more patients in the lixisenatide groups achieved an HbA_1c_ < 7.0% (53 mmol/mol; ranging from 47 to 69% with once daily dosing and from 51 to 77% with twice daily dosing), compared with 32% of those in the placebo group (*P <*0.05) at week 13 (Fig. 2). Further improvement in glycaemic control to HbA_1c_ < 6.5% (48 mmol/mol) at study end was observed in significantly more patients in the lixisenatide groups than in the placebo group (7.5%); one-third of patients receiving 20 or 30 μg once daily and 5, 10 and 20 μg twice daily achieved this goal (*P <*0.0001 for all of these groups vs. placebo and *P* = 0.0315 vs. placebo for 5 μg once daily; Fig. 2).

As noted in Table 2, there were dose-dependent reductions from baseline in fasting plasma glucose and also in daily averaged seven-point self monitored blood glucose, 2 h post-prandial plasma glucose concentrations, and in body weight with lixisenatide.

**Table 2:** The least square adjusted mean ± sem changes in fasting plasma glucose, daily averaged self-monitored seven-point blood glucose and 2 h postprandial plasma glucose following 13 weeks of treatment with lixisenatide once daily or twice daily according to dosage and regimen in the ITT population

		Lixisenatide							
		
	Placebo (*n* = 108)	5 μg QD(*n* = 55)	10 μg QD(*n* = 51)	20 μg QD(*n* = 53)	30 μg QD(*n* = 52)	5 μg BID(*n* = 51)	10 μg BID(*n* = 54)	20 μg BID(*n* = 52)	30 μg BID(*n* = 53)
Fasting plasma glucose (mmol/l)	−0.21 ± 0.19	−0.62 ± 0.24	−0.54 ± 0.25	−0.80 ± 0.25	−1.02 ± 0.25*	−0.19 ± 0.24	−0.98 ± 0.24*	−1.13 ± 0.25*	−1.42 ± 0.25*
Average self-monitored seven-point blood glucose (mmol/l)	−0.53 ± 0.18	−1.23 ± 0.24*	−1.27 ± 0.24*	−1.74 ± 0.24*	−1.77 ± 0.25*	−0.88 ± 0.24	−1.60 ± 0.24*	−1.83 ± 0.24*	−2.08 ± 0.24*
2 h postprandial glucose (mmol/l)^a^	−0.41 ± 0.46	−2.12 ± 0.67†	−3.57 ± 0.62*	−3.65 ± 0.68*	−4.33 ± 0.71*	−2.01 ± 0.61†	−3.51 ± 0.62*	−4.12 ± 0.68*	−4.61 ± 0.68*
Body weight (kg)	−1.94 ± 0.32	−2.00 ± 0.40	−2.39 ± 0.42	−3.01 ± 0.41*	−3.47 ± 0.41*	−2.10 ± 0.41	−2.21 ± 0.41	−2.61 ± 0.41	−3.89 ± 0.41*

The ITT population consisted of all randomized patients who took at least one dose of the study medication and had a baseline and at least one post-baseline on-treatment value for efficacy variables.

* *P* < 0.01

† *P* < 0.05 in the step-down linear trend test.

a In a subgroup of patients undergoing standardized breakfast in selected sites (approximately half of total sites). The standardized 500 kcal breakfast, which consisted of orange juice (180 ml), toasted bread (60 g), jam or preserves (20 g), butter or margarine (10 g), whole milk (120 ml) and coffee or tea with non-nutritive sweetener (if desired), was consumed within a 15 min period.

Data are mean ± SEM.

### Safety and tolerability

The most frequent adverse events were gastrointestinal, primarily dose-dependent nausea (Table 3). The onset of gastrointestinal adverse reactions was observed during the first 5 weeks of the study in the majority of cases, and these were usually mild-to-moderate in intensity. No cases of pancreatitis were experienced. There was no evidence of a dose relationship with symptomatic hypoglycaemic episodes [ranging from 1 to 3 events (0.9–5.7%) per group), which were mostly mild in intensity. No patients experienced severe hypoglycaemia.

**Table 3:** Number (%) of patients with treatment-emergent adverse events occurring in ≥ 10% in any one group and symptomatic hypoglycaemia in the safety population

		Lixisenatide							
		
Type of adverse event	Placebo (*n* = 109)	5 μg QD (*n* = 55)	10 μg QD (*n* = 52)	20 μg QD (*n* = 55)	30 μg QD (*n* = 54)	5 μg BID(*n* = 53)	10 μg BID (*n* = 56)	20 μg BID (*n* = 54)	30 μg BID (*n* = 54)
Any treatment-emergent adverse events	65 (59.6)	31 (56.4)	26 (50.0)	37 (67.3)	42 (77.8)	30 (56.6)	32 (57.1)	38 (70.4)	40 (74.1)
Any serious treatment-emergent adverse events	3 (2.8)	0	1 (1.9)	1 (1.8)	3 (5.6)	0	1 (1.8)	2 (3.7)	0
Nausea	5 (4.6)	4 (7.3)	6 (11.5)	14 (25.5)	19 (35.2)	4 (7.5)	8 (14.3)	12 (22.2)	18 (33.3)
Vomiting	1 (0.9)	2 (3.6)	3 (5.8)	3 (5.5)	10 (18.5)	3 (5.7)	4 (7.1)	5 (9.3)	2 (3.7)
Diarrhoea	8 (7.3)	3 (5.5)	4 (7.7)	5 (9.1)	4 (7.4)	3 (5.7)	4 (7.1)	6 (11.1)	14 (25.9)
Headache	11 (10.1)	7 (12.7)	3 (5.8)	7 (12.7)	7 (13.0)	7 (13.2)	5 (8.9)	6 (11.1)	4 (7.4)
Dizziness	7 (6.4)	1 (1.8)	4 (7.7)	4 (7.3)	6 (11.1)	3 (5.7)	5 (8.9)	2 (3.7)	5 (9.3)
Symptomatic hypoglycaemia	1 (0.9)	1 (1.8)	2 (3.8)	1 (1.8)	1 (1.9)	3 (5.7)	1 (1.8)	3 (5.6)	1 (1.9)

Data are *n* (%). Treatment-emergent adverse events were defined as adverse events that developed or worsened during the on-treatment period (the time from the first dose of study medication up to 3 days after the last dose). The safety population was composed of all randomized patients who took at least one dose of the study medication during the double-blind treatment phase.

There were zero to three (5.6%) serious adverse events in the lixisenatide groups and three (2.8%) in the placebo group (Table 3). These events included one patient in the lixisenatide 30 μg once daily group who discontinued treatment owing to a few seconds of loss of consciousness and one in the lixisenatide 10 μg once daily group who discontinued secondary to an allergic reaction (a 30 min episode of pruritus over the entire body within 10 min of injecting study drug after 3 weeks of study treatment, and a second episode 10 min after the next injection, given 3 days later, with swollen lips/tongue and difficulty in breathing that resolved within minutes of receiving an oral antihistamine). Two non-serious cases of urticaria were reported with lixisenatide and three with placebo. There was evidence for a relationship between the lixisenatide dose and frequency of adverse events (mainly due to gastrointestinal adverse events), but not with the number of serious adverse events (Table 3).

The frequencies of patient discontinuations from the study due to treatment-emergent adverse events ranged from 1.8 to 11.1% in the once daily lixisenatide groups and from 0 to 14.8% in the twice daily lixisenatide groups, while 1.8% of patients taking placebo discontinued.

No clinically significant changes were detected by laboratory safety assessments and on 12-lead electrocardiogram. Mean systolic and diastolic blood pressure levels were 130/80 mmHg, respectively, at baseline. A clear trend of systolic and diastolic blood pressure reductions from baseline to end-point occurred with each lixisenatide dose (ranging from −2 to −9 mmHg for the systolic, and −2 to −4 mmHg for the diastolic blood pressures), and also with placebo (−3 and −2 mmHg for systolic and diastolic blood pressures, respectively). The apparent reductions in blood pressure were observed as early as week 1 in most of the groups and, therefore, appeared to be independent of reductions in HbA_1c_ and body weight. There were no relevant changes in heart rate from baseline to end-point in any of the groups.

The percentages of anti-lixisenatide antibody-positive subjects at end-point ranged from 43.1% in the 10 μg once daily group to 71.2% in the 20 μg twice daily group. No relevant differences were observed in terms of safety and efficacy between the patient populations with antibody-positive and negative status at study-end for all dose regimens.

## Discussion

In this study, the new GLP-1 receptor agonist lixisenatide significantly improved glycaemic control from a mildly elevated mean baseline HbA_1c_ [∼7.55% (∼59.0 mmol/mol)] in patients with Type 2 diabetes mellitus inadequately controlled with metformin.

A total of eight regimens, comparing four doses each administered once or twice daily, were compared with placebo in order to characterize fully the dose–effect profile of lixisenatide when added to previous metformin monotherapy. At week 13, statistically significant reductions in the primary end-point—the HbA_1c_ level—were observed for each dose of lixisenatide. The efficacy of lixisenatide was dose related across the once daily dose range with regard to improvements in the primary and secondary end-points of fasting plasma glucose, daily averaged seven-point self monitored blood glucose and 2 h post-prandial plasma glucose. Notably, the once daily and twice daily lixisenatide regimens achieved similar levels of efficacy, and doubling the daily dose (in the twice daily regimens) did not provide relevant additional improvements in glycaemic control over the once daily regimens. The efficacy of lixisenatide appeared to reach a plateau at a dose of 20 μg once daily, with further increases offering limited benefit relative to the increase in drug exposure. This is in accordance with a pharmacodynamic study that found that both 20 μg once daily and 20 μg twice daily of lixisenatide significantly improved HbA_1c_ to a similar extent vs. placebo, despite the short (4 week) treatment period [13].

There have been a few previous dose-ranging studies of exenatide or liraglutide [16–19]. A Phase II dose-ranging study of exenatide (2.5–10.0 μg) was performed over 4 weeks [18]. Initial dose-ranging studies of liraglutide monotherapy evaluated doses of 0.045–0.75 mg once daily over 12 weeks [16,17], with the highest doses giving HbA_1c_ reductions similar to that observed with lixisenatide 20 μg once daily in the present study, but from a higher baseline HbA_1c_ [16]. A subsequent study appeared to establish a dose–effect plateau at 1.25 and 1.90 mg once daily in patients with poorer glycaemic control at baseline [HbA_1c_ 8.1–8.5% (65–69 mmol/mol)] [19].

Importantly, over two-thirds of patients on lixisenatide 20 μg once daily and 30 μg once daily reached the target of HbA_1c_ < 7.0% (53 mmol/mol), compared with 32% of those taking placebo. This suggests that lixisenatide could be a useful option for helping the large fraction of patients in clinical practice who do not achieve recommended HbA_1c_ goals [20], as well as for overcoming the limited therapeutic response provided by some existing therapies when the baseline is mildly elevated.

Of note, improvements in glycaemic control with lixisenatide were coupled with reductions in body weight. This is an important finding in light of the high prevalence of obesity and overweight in this population and the relationship of weight with insulin resistance and cardiovascular disease [21], as well as the tendency of current intensive therapy to cause weight gain [22].

Overall, lixisenatide was well tolerated. Consistent with other GLP-1 receptor agonists [16,17,23–25], gastrointestinal adverse events were the most common with lixisenatide, and nausea was the most frequent of these. Nevertheless, it appears that fewer patients experienced nausea with the 20 μg once daily dose than figures previously reported in clinical studies of twice-daily exenatide [23–25], but only head-to-head studies can substantiate this suggestion. The onset of nausea occurred predominantly during the first half of the study and was mild to moderate in intensity. Only one patient discontinued the study due to nausea (and none for vomiting) in the 20 μg once daily lixisenatide group. The risks of hypoglycaemia and serious adverse events were low and similar across the lixisenatide dose range. Based on these data, a dose of 20 μg once daily appears to balance maximal efficacy with good tolerability. However, the present study has the limitation of a relatively short treatment period (13 weeks), and the full long-term effect of lixisenatide on glycaemic control and body weight remains to be determined.

In conclusion, in this thorough dose-ranging study of four doses and two regimens, lixisenatide significantly improved glycaemic control in mildly hyperglycaemic patients previously on metformin monotherapy, with associated weight loss and without causing significant hypoglycaemia. Clear dose–response relationships and similar levels of efficacy were seen for the once daily and twice daily regimens, with a 20 μg once daily dose showing the best efficacy-to-tolerability ratio. This new, once-daily GLP-1 receptor agonist shows promising efficacy, safety and tolerability in the management of Type 2 diabetes, but further investigations in long-term studies are needed.

## Investigators for the DRI6012 Study Group

*Brazil* Antonio Chacra, Marco Antonio Dias, Mauro Dinato, Freddy Eliaschewitz, Vivian Ellinger, Adriana Forti, Amelio Godoy, Marilia Gomes, Alfredo Halpern, Miguel Hissa, Antonio Carlos Lerario, Geisa Macedo, Euler Manenti, Jose Egidio Oliveira, Hermelinda Pedrosa, Nelson Rassi, Giuseppe Repetto, Joao Salles, Jose Augusto Sgarbi, Ruy Lyra da Silva Filho, Henrique Suplicy, Balduino Tschiedel, Rosa Vargas and Maria Zanella.

*Canada* Ronnie Aronson, James Cha, Ronald Goldenberg, Neil Hudson, Mustafa Kamouna, Lisa Klassen, Jacobus Kooy, Randy MacKinnon, Karl Misik, Michael O’Mahony, Alexander Skamene, Yaw Twum-Barima, Anthony Wade and Vincent Woo.

*Poland* Marek Konieczny, Jerzy Kuleta, Grzegorz Laskawiec, Piotr Mader, Adam Pieniazek, Marek Szolkiewicz, Ireneusz Szymczyk and Danuta Zytkiewicz-Jaruga.

*Romania* Alexandru Barnea, Mihaela Busegeanu, Carmen Crisan, Radu Lichiardopol, Nicoleta Mindrescu, Magdalena Morosanu, Lavinia Pop, Milivoi Stamoran and Iosif Szilagyi.

*Russian Federation* Olga Barbarash, Nina Chernova, Galina Chumakova, Tatjana Raskina and Elena Zonova.

*Ukraine* Volodymyr Botsyurko, Vitaliy Katerenchuk, Boris Mankovsky, Tetyana Pertseva, Alexander Serhiyenko and Maryna Vlasenko.

*USA* John Abernethy, Andrew J. Ahmann, Richard F. Arakaki, Suresh Baliga, Anthony J. Bartkowiak Jr, Bantwal Richard Bernstein, Loray Blair-Britt, Michael Bolognese, Robert S. Busch, Robert Buynak, LeRoy J. Byrd, David W. Cardona, Christopher M. Chappel, Richard B. Christensen, Lisa Cohen, Terry Nizo Copeland, Clinton Corder, Yvette Crabtree, Arcot Dwarakanathan, Daniel S. Donovan, H. Jackson Downey, Robert M. Evans, Robert Eyzaguirre, Ronald B. Goldberg, Barry Jay Goldstein, G. M. Gollapudi, Gregory M. Gottschlich, Michael Guice, Yehuda Handelsman, Israel Hartman, John Arthur Hoekstra, Priscilla Hollander, Robert L. Jackson, David Jasper, Marvin Kalafer, William A. Kaye, Lawrence R. Koehler, Dan Lender, Peter Lodewick, Charles F. Lovell, Kathryn Jean Lucas, Timothy J. Lyons, Marco Marcelli, Wendell Miers, Sam S. Miller, Paul S. Norwood, Fernando Ovalle, Phillip J. Peters, Fatima Phillips, Brian R. Riveland, Syed W. Rizvi, Julio Rosenstock, Douglas R. Schumacher, Sherwyn L. Schwartz, Robert B. Schwartz, Cranford L. Scott, Anuja Jasvant Shah, Loknath Shandilya, Mansur Shomali, Mohammad I. Siddiqqi, Jeremy Soule, Robert Strzinek, Danny Sugimoto, James Underberg, Thomas A. Wade, Bret A. Wittmer, Berto M. Zamora and Howard C. Zisser.

## Abbreviations

DPP-4: dipeptidyl-peptidase-4
GLP-1: glucagon-like peptide-1
IFCC: International Federation of Clinical Chemistry and Laboratory Medicine
NGSP: National Glycohemoglobin Standardization Program
QD: once daily
